# Elderly People’s Acceptance of Death: A Study of a Polish Cohort

**DOI:** 10.3390/ijerph16183374

**Published:** 2019-09-12

**Authors:** Mariusz Wysokiński, Wiesław Fidecki, Magdalena Jarosz

**Affiliations:** Department of Basic Nursing and Medical Teaching, Chair of Development in Nursing, Faculty of Health Sciences, Medical University of Lublin, 20-059 Lublin, Poland

**Keywords:** elderly people, death, acceptance

## Abstract

**Introduction:** Old age is usually the natural time for people to prepare for death, which may evoke various emotions ranging from acceptance to hostility. **Aim of the work:** The study aimed at specifying various degrees to which elderly people accept death. **Material and method:** The study employed the diagnostic poll method and an Inventory of the Attitude towards Death (IAD) poll questionnaire. The investigation was administered in a cohort of 150 people over 65 years of age living in Poland. **Results:** The highest results were noted both for males and females on the “Value” scale (M = 4.94 and M = 4.96) and on the “Necessity” scale (M = 4.79 and M = 4.95). These two scales also had the highest values in the cohorts of city dwellers and country dwellers. A statistically significant difference (Z = 2.339, *p* = 0.019) was found in the “Necessity” dimension between investigated people with higher education and others. Furthermore, statistically significant differences were found in the following dimensions: “Mysteriousness”, “Value”, “Dread”, “Tragedy”, and “Absurdity”. Comparing death dimensions in people with chronic illnesses and in those without such illnesses, meaningful statistical differences were noted in the “Necessity” dimension (t = 1.983, *p* = 0.049). However, analysing death dimensions in people who suffered because of a severe illness in a family member and respondents whose families were healthy, statistically significant differences were noted in the “Absurdity” dimension (t = 2.057, *p* = 0.041). **Conclusions:** Sex, the place of residence, and death of a close person did not affect elderly people’s acceptance of death. On the other hand, those suffering from chronic diseases were more aware of the inevitability of death. People without higher education were also more aware of the inevitability of death. Suffering of a serious disease of a close one considerably affected acceptance of death in the elderly.

## 1. Introduction

Death is a serious aspect of a human life. On the one hand, its presence is relatively common and natural in lives of all people, on the other hand, it is connected with angst and anxiety in many [1]. Death is closely connected with old age as this time of life physiologically prepares a lot of people for leaving this world. Nevertheless, death is often a taboo subject. People are afraid to mention it and are thus unwilling to discuss the very phenomenon of death. Thinking about it might result in fear, anxiety, and uncertainty [1,2]. Attitudes to death are conditioned by numerous factors: culture, religion, lifestyle, environmental conditions, and access to health services [3]. This brings attitudes towards death in elderly people and death-related problems in focus for scientific research. The scope of analysis included anxiety about death resulting from age, psychological and physical condition, the feeling of happiness, hopelessness, as well as the influence of the place of abode, social support received, ethnic background, the sense of fulfilment and related emotions [3,4,5,6,7,8]. Many studies focus their analyses on cognitive and emotional reactions connected with the phenomenon of death, e.g., fear and anxiety [9,10,11]. However, the findings are often diversified and inconclusive, which may result from the need to include into analysis the subject’s cultural background, their religion, stability of their world view and resulting practice. [12,13]. This shows that perceiving death is often highly individualised and specific to one community. Most studies have been performed in the socio-cultural conditions of highly developed countries, and unfortunately, only few have been done in the context of transforming Eastern European countries, e.g., Poland. People from this region have recently been highly devoted to their cultural traditions and practising multigenerational family rites. These factors have been shaping their death-related attitudes, which, according to Kaczmarek, could take the following forms. The first is indifference, i.e., a person denies death in their consciousness, treating it as a destruction. This negative attitude connects the very moment of death with both physical and mental loneliness. Death might be approached differently when looked at in the theological context with a perspective of an eternal life. Finally, it might be claimed that death should be neither brought forward nor postponed. The last approach expresses an acceptance of death through familiarisation. Presently, there are a number of centres (e.g., hospices) that make a human being ready for leaving this world [14]. Szaniawski enumerates five attitudes towards death that are conditioned by a human personality: religious, ambivalent, peaceful, evasive, and terrified [15]. However, Makselon enumerates six attitudes, presenting them as value types: economic, theoretical, social, political, aesthetic, and religious [16]. urbanisation, changes in the family structure, changes in culture, transformations of local groups, secularisation, and medical progress trigger changes in the general attitude towards death and its acceptance in the inhabitants of Poland [17]. 

This study aims at specifying levels of elderly people’s acceptance of death. 

## 2. Materials and Methods 

The investigation employed the diagnostic poll method with the questionnaire technique. The Inventory of the Attitude towards Death (IAD) poll questionnaire by J. Makselon was the major research tool, which consists of three parts. The first is a scale that includes 33 questions to be answered by means of statements ranging from 1 to 7. The second part is comprised of a questionnaire whose aim is to probe three aspects of the attitude towards death:

1. the attitude towards death as a general phenomenon
2. death of a close person
3. a possibility of respondent’s death.

Each of the aforementioned aspects features four sentences that refer to identical processes or psychological states, however, they present the attitude in different ways. The third part consists of nine unfinished sentences based on the projection psychology. The scale allows to examine attitudes towards death: necessity, value, centrality, mysteriousness, dread, tragedy, destructiveness, and absurdity [16]. Necessity stems from becoming aware of death being an irrevocable phenomenon in nature. Centrality refers to a level of being interested in death and pondering it. Mysteriousness results from perceiving it as a nagging unanswerable question, the greatest enigma accompanied by anxiety. Value may contribute to a better understanding of oneself and immunize one to health and psychological problems that may occur. Furthermore, it can also influence one to make corrections to their current lifestyle. Dread is connected with death causing fear, anxiety and physical pain. When it comes to the aspect of Tragedy, death is seen as a shocking and unlucky event that causes despair. Destructivity refers to death destroying any opportunities for development that restricts looking for some sense in life, something that demobilizes human activities. Absurdity is connected with generating a feeling of pointlessness and senselessness of existence [18]. All of these aspects allow to evaluate attitudes towards death in a general, abstract sense, avoiding the focus on selected elements, e.g., fear and an influence on personality. 

Reliability of a scale calculated as a coefficient of its internal compatibility is 0.74. A coefficient of stability, i.e., so-called absolute stability for specific dimensions of the scale ranged from 0.66 to 0.84 [19].

The investigation was administered in compliance with the Helsinki Declaration. 

Values of the analysed parameters were characterised by means of the cardinality and the percentage or a mean value and a standard deviation. The U-Mann–Whitney test and the t-student test were used to assess differences between the two groups for continuous variables. 5% error of inference and the connected significance level of *p* < 0.05 were adopted that pointed to the existence of statistically significant differences. Statistical investigation was administered on the basis of the STATISTICA 10.0 (StatSoft, Kraków, Poland) software. 

The research involved people over 65 years of age without diagnosed dementia changes who were staying in their home environment, and who were not suffering from acute stages of an illness, and were not in the terminal phase of a disease. The study involved the cohort of 150 people over 65 years of age. The mean age was 67.4. Females predominated, comprising 56.67% (*n* = 85) of the cohort, similarly, city dwellers comprised 59.3% (*n* = 89), widows or widowers 37.3% (*n* = 56), those declaring education other than higher comprised 82.0% (*n* = 123) and 54.7% (*n* = 82) declared they were not suffering from any chronic diseases (Table 1). 

## 3. Results

Using the IAD scale allowed to determine eight dimensions of the attitude towards death. The highest results for females were noted in the “Value” scale (M = 4.94) and in the “Necessity” scale (M = 4.79). In case of males, the highest values were noted in the “Necessity” dimension (M = 4.95) and the “Value” dimension (M = 4.95). No significant differences were found between investigated males and females on the IAD scale. Accounting for the residence of investigated people, city dwellers noted highest results for the “Dimension” value (M = 4.99) and for the “Necessity” value (M = 4.77). Very similar results were noted for the country dwellers. In this case, the dimensions of “Necessity” (M = 5.00) and “Value” (M = 4.89) also had the highest mean values. No statistically significant values were noted in the attitude towards death between city dwellers and country dwellers. 

Statistically significant differences were noted in the “Necessity” dimension (Z = 2.339, *p* = 0.019) analysing results of the dimension of death in people with higher education and in respondents with other types of education In this dimension, it is the group of respondents with education other than higher that scored more (M = 4.94). Comparing dimensions of death in people with chronic illnesses and without such conditions, statistically significant differences were noted in the “Necessity” dimension (t = 1.983, *p* = 0.049). The group of chronically ill people scored higher (M = 5.02) in this dimension. 

However, analysing results of death dimensions in those who suffered through witnessing a chronic disease in a close person and those from healthy families, statistically significant differences were found in the “Absurdity” dimension (t = 2.057, *p* = 0.041). Here, the group of respondents whose close families did not suffer from chronic illnesses scored higher (M = 3.73). 

Taking recent death of a close family member into account, the highest results in the group in which a close family member had died recently were noted for the “Value” dimension (M = 4.93) and the “Necessity” dimension (M = 4.84). Very similar results were scored by respondents who had not recently suffered from death of a family member. In this case, the dimensions of “Necessity” (M = 4.94) and “Value” (M = 5.00) also obtained the highest mean results (Table 2). 

## 4. Discussion

The final stage of a human life, i.e., the old age, is a time when many people to account for their whole life. Many elderly people pay special attention to human life’s sense and purpose. The subject of death and dying becomes ever closer. For some, it becomes a taboo which they would rather not touch, however, others find it a natural topic that causes neither anxiety nor fear [6]. There is always a certain diversity in attitudes towards life and death, which is caused by differences in lifestyle, culture, the general outlook, satisfaction with life and the place of residence [20,21,22]. 

Research into attitudes towards death involved the cohort of 150 geriatric people. Analysing IAD results—the scale distinguishing eight dimensions of attitudes towards death accounting for respondents’ sex—hardly any statistically significant differences were found between males and females. The cohorts comprised of males and females scored the highest results in the “Value” dimension (respectively, females M = 4.94; males M = 4.95). Both groups also scored high in the “Necessity” dimension (females M = 4.79, males M = 4.96). This suggests they were aware of the inevitability. This might result from the fact the older a person is, the more often they have to deal with the death of their relatives, and, consequently, they more often reflect on passing away and its inevitability. The abovementioned results were reflected in the division of attitudes towards death according to Kaczmarek. He points out that one of the possible attitudes was constituted by accepting death through getting familiar with thinking about it and being aware if its inevitability. Another attitude consisted in approaching death with a hope for eternal life [14]. Such attitude might result from the fact that research involved people who had not fully experienced the phenomenon of “old age commercialisation” shaped by the capitalist system, and their life attitudes were conditioned by determinants other than “to have” [23]. 

No significant statistical differences between city dwellers and country dwellers were noted on the basis of the IAD scale accounting for respondents’ place of residence. The highest scores were obtained for the “Value” dimension (city M = 4.99; country M = 4.89) and “Necessity” (city = M 4.77; country M = 5.00), which was the same as in the case of respondents’ sex. “Centrality” dimension obtained the lowest mean value (city M = 2.52; country M = 2.73). This means that death did not occupy the central position in respondents’ lives. This approach might be caused by sociocultural changes. Seniors more and more often actively organise their retirement; they frequently travel, participate in Third Age University activities and are active in non-government organisations. These might be considered to be substitute activities that arise as a result of departing from the traditional social model that used to help to get familiar with the awareness of death e.g., in multi-generational families. However, such new forms of activity seem to comply with the offer by the mass culture, i.e., promoting vitality, consumer lifestyle, treating death as a banal media fact, and expecting death to be quick and painless, certainly not preceded with a prolonged illness. However, Dąbrowski presents a different point of view claiming there is a considerable difference between city and country dwellers in their perceptions of death. He points to the fact that the phenomenon of agglomeration causes denial and rejection of the notion of death and all things that might be connected with it [18]. 

The authors’ own research unveiled statistical differences in the “Necessity” dimension between people with higher education and respondents with other types of education. Higher results were scored by the group of respondents with education other than higher, i.e., these respondents were more aware of the inevitability of death as the final stage of their lives. This may result from the fact that many people with higher education frequently defy the mystical aspect of death which results from social and cultural traditions and instead try to perceive it more rationally, or even reduce it to its purely biological sense. This often trivialises death and indirectly leads to avoiding conversations focusing on this topic. Differences are confirmed and explained by Szaniawski who described attitudes towards death conditioned by personality. One of such attitudes is the religious one, which he explains causes adopting death as a reconciliation. People adopting a religious perspective experience fear of death less intensively This means that they usually find it much easier to accept death irrespectively of their religion [4,15,23]. Additionally, religion may be a factor that restricts the risk of suicidal behaviours in elderly people [3]. 

Administered investigations show statistically significant differences in the “Necessity” dimension between those suffering from a chronic disease and healthy respondents. Chronically ill respondents had higher results as they were more aware of the inevitability of death being the final stage of their lives. 

Results of own research showed statistically significant differences in the “Absurdity” dimension between people suffering from witnessing a serious illness of a family member and respondents whose families were healthy. This means such respondents perceived death as the absurdity of life and the end of hope. Other authors pointed out to religion as a factor that facilitated acceptance of death in a chronic disease [24]. 

Analysing attitudes towards death and the meaning of recent death of a family member, no statistically significant results were discovered. In both groups, the highest results were obtained in “Value” and “Necessity” dimensions. This means that respondents found death to be a moment of truth about themselves. They often thought about both the death of their family members and their own, and they were aware of its inevitability. Lowest results were obtained in the “Centrality” dimension, which means that death did not occupy a central place in the lives of those people, albeit they remembered about its inevitability. 

The authors are aware of research limitations resulting mostly from the insignificant size of the study cohort and the choice of the research tool. However, one must remember that acceptance of death is conditioned not only by individual factors, but also by socio-cultural ones and therefore using research tools accounting for specific nature of a given community seems justifiable. Analysing the phenomenon of death, most authors mainly focus on the elderly in life threating situations and terminal diseases but fail to analyse this phenomenon when such a threat is absent. One must also remember that Europe is culturally and socially diverse, and for example there are differences between Poland where most people are Christian, and the Czech Republic where the majority of the population are atheists. Furthermore, the situation in Germany and France is different as these countries are religiously diverse.

## 5. Conclusions

In conclusion, the findings indicate that in studied cohort of elderly people: no differences were discovered in acceptance of death accounting for sex, place of residence and a death of a family member; chronically ill people have a higher awareness of the inevitability of death as the final stage of their lives; people with education other than higher were more aware of the inevitability of death; and finally, Suffering because of witnessing an illness in a family member can affect the acceptance of death in elderly people. 

## 6. Practical Postulates

Research findings prove there is are factors that are key to affecting many people’s perception of death. Healthcare workers might be able to control some of these factors in healthcare institutions and in a senior’s home environment. It is, for example, possible to seek practices forms that might reduce some people’s fears connected with death. Such forms need to account for sociocultural conditions in a given population, but they may also tap into self-esteem, building support groups and finding suitable mentors.

## Figures and Tables

**Table 1:** Characteristics of the investigated cohort.

Variable	Category of the Variable	Number (*n*)	Percentage (%)
Sex	Female	85	56.67
	Male	65	43.33
Residence	City	89	59.30
	Country	61	40.70
Marital status	Single	23	15.30
	Married	55	36.70
	Divorced	16	10.70
	Widow/widower	56	37.30
Education	Other than Higher *	123	82.00
	Higher **	27	18.00
Chronic illness	Yes	68	45.30
	No	82	54.70

* education other than higher certified with a diploma (elementary, vocational, secondary); ** higher education i.e., respondents are diploma-holding graduates.

**Table 2:** Dimensions of attitudes towards death in the investigated cohort of seniors.

Variable	Variable Category		Necessity (Ne)	Centrality (Ce)	Mysteriousness (My)	Value (Va)	Dread (Dr)	Tragedy (Tr)	Destructiveness (De)	Absurdity (Ab)
Sex	Female	M	4.79	2.63	4.04	4.94	4.37	4.33	4.02	3.44
		SD	0.93	0.94	1.20	0.94	0.98	1.16	1.04	1.21
	Male	M	4.96	2.56	3.98	4.95	4.33	4.31	4.22	3.59
		SD	0.78	0.96	1.20	1.05	0.94	1.12	1.00	1.18
Statistical analysis		t	−1.141	0.429	0.257	−0.066	0.264	0.082	−1.159	−0.810
		*p*	0.256	0.669	0.789	0.948	0.792	0.934	0.248	0.419
Residence	City	M	4.77	2.52	3.89	4.99	4.29	4.24	4.03	3.46
		SD	0.88	0.89	1.16	1.04	1.00	1.17	1.06	1.23
	Country	M	5.00	2.73	4.19	4.89	4.45	4.44	4.21	3.57
		SD	0.84	1.02	1.23	0.90	0.89	1.09	0.89	1.14
Statistical analysis		t	−1.677	−1.333	−1.516	0.614	−1.048	−1.007	−1.074	−0.541
		*p*	0.096	0.184	0.132	0.540	0.296	0.316	0.284	0.589
Education	Higher	M	4.53	2.58	4.23	4.81	4.34	4.10	4.15	3.64
		SD	0.79	1.00	1.08	1.12	1.03	1.24	1.08	1.19
	Other than higher	M	4.94	2.61	3.96	4.98	4.36	4.37	4.09	3.47
		SD	0.87	0.94	1.22	0.95	0.95	1.12	1.02	1.20
Statistical analysis		Z	2.339	0.142	−1.005	0.429	0.208	1.141	0.108	−0.833
		*p*	0.019	0.886	0.315	0.668	0.835	0.253	0.914	0.405
Own chronic illness	Yes	M	5.02	2.49	4.00	4.97	4.34	4.39	4.08	3.46
		SD	0.85	1.01	1.12	0.91	0.94	1.07	1.02	1.18
	No	M	4.74	2.69	4.03	4.93	4.37	4.27	4.12	3.54
		SD	0.87	0.89	1.26	1.05	0.98	1.20	1.03	1.21
Statistical analysis		t	1.983	−1.319	−0.170	0.285	−0.188	0.639	−0.229	−0.407
		*p*	0.049	0.189	0.865	0.776	0.851	0.524	0.819	0.685
Chronic illness of a family member	Yes	M	4.95	2.65	4.06	5.00	4.36	4.28	4.04	3.33
		SD	0.81	0.97	1.18	0.86	0.93	1.14	1.06	1.07
	No	M	4.75	2.54	3.94	4.87	4.34	4.38	4.19	3.73
		SD	0.94	0.92	1.21	1.14	1.00	1.15	0.98	1.32
Statistical analysis		t	−1.351	−0.672	−0.623	−0.835	−0.117	0.540	0.908	2.057
		*p*	0.179	0.502	0.534	0.405	0.907	0.590	0.365	0.041
Death of a family member	Yes	M	4.84	2.61	4.09	4.93	4.40	4.38	4.16	3.60
		SD	0.90	0.96	1.19	0.95	0.96	1.15	1.07	1.17
	No	M	4.94	2.55	3.74	5.00	4.18	4.12	3.92	3.17
		SD	0.76	0.93	1.17	1.12	0.93	1.12	0.85	1.22
Statistical analysis		Z	0.524	−0.312	−1.362	0.211	−1.286	−1.075	−1.000	−1.570
		*p*	0.600	0.754	0.173	0.832	0.198	0.282	0.317	0.116

M—mean, SD—standard deviation, Z—result of U Mann-Whitney’s test, t—result of t-Student’s test, *p*—statistical significance.
